# Model of local hydrogen permeability in stainless steel with two coexisting structures

**DOI:** 10.1038/s41598-021-87727-5

**Published:** 2021-04-20

**Authors:** Akiko N. Itakura, Naoya Miyauchi, Yoshiharu Murase, Taro Yakabe, Masahiro Kitajima, Satoka Aoyagi

**Affiliations:** 1 National Institute for Materials Science, 1-2-1 Sengen, Tsukuba, Ibaraki 305-0047 Japan; 2 Seikei University, 3-3-1 Kitamachi, Kichijoji, Musashino, Tokyo 180-8633 Japan

**Keywords:** Materials science, Physics

## Abstract

The dynamics of hydrogen in metals with mixed grain structure is not well understood at a microscopic scale. One of the biggest issues facing the hydrogen economy is “hydrogen embrittlement” of metal induced by hydrogen entering and diffusing into the material. Hydrogen diffusion in metallic materials is difficult to grasp owing to the non-uniform compositions and structures of metal. Here a time-resolved “operando hydrogen microscope” was used to interpret local diffusion behaviour of hydrogen in the microstructure of a stainless steel with austenite and martensite structures. The martensite/austenite ratios differed in each local region of the sample. The path of hydrogen permeation was inferred from the time evolution of hydrogen permeation in several regions. We proposed a model of hydrogen diffusion in a dual-structure material and verified the validity of the model by simulations that took into account the transfer of hydrogen at the interfaces.

## Importance of hydrogen visualization in the hydrogen economy

With an eye to the arrival of the hydrogen economy, many researchers are actively working on materials science; for example, researching tough materials resistant to “hydrogen embrittlement”, a degradation that can be induced in metals by hydrogen entering from outside and diffusing into the material. However, hydrogen diffusion in metallic materials is difficult to grasp owing to the non-uniform compositions and structures of metals. For example, stainless steels in practical use generally have complex structures consisting of partially or fully austenite, ferrite, and martensite phases, each with their phase boundaries, precipitates, internal strains, and so on. Elucidating the behaviour of hydrogen in these individual phases is very important for the purpose of controlling hydrogen in metals. Hydrogen permeation can be controlled by identifying local pathways of hydrogen and blocking the pathways. However, these matters have not been clarified well, because it is difficult to visualize hydrogen.

Electron-stimulated desorption (ESD)—a popular technique in the field of surface science to study adsorption or desorption physics or to identify adsorbates—is a powerful investigative tool for direct detection of hydrogen in a metal^1–3^, usually without causing damage. To visualize the two-dimensional distribution of hydrogen on a surface, we have constructed an original “operando hydrogen microscope”, which is an apparatus using ESD coupled with a hydrogen supply system. With this system, images of hydrogen at the surface of many materials can be created, and the signals can be integrated over time. In previous studies, we have used this system to acquire maps of hydrogen permeating from the bottom surface of a sample to the top surface, with continuous measurement over 3 days; we have measured the diffusion coefficients of austenite-dominant regions and martensite-dominant regions in stainless steel based on changes in the amounts of hydrogen permeating over time^4–6^.

In the current study, we measured hydrogen permeation in several local regions of a sample of cold-worked dual-phase SUS304 stainless steel, with each region having a different martensite/austenite ratio. Electron backscattering diffraction (EBSD) was used to determine the martensite/austenite ratio of each region, which ranged from 3 to 76%. By quantitatively calculating the diffusion coefficient in each region, we were able to create a model of hydrogen diffusion through a dual-phase region. To the best of our knowledge, there are a few study has created models and carried out simulations of hydrogen diffusion that consider the effect of dual-phase materials in micro-scale^7–10^. Below, we discuss our permeation model and the experimental results of local hydrogen permeation.

## Detecting the time dependence of local hydrogen permeation

**Figure 1:**
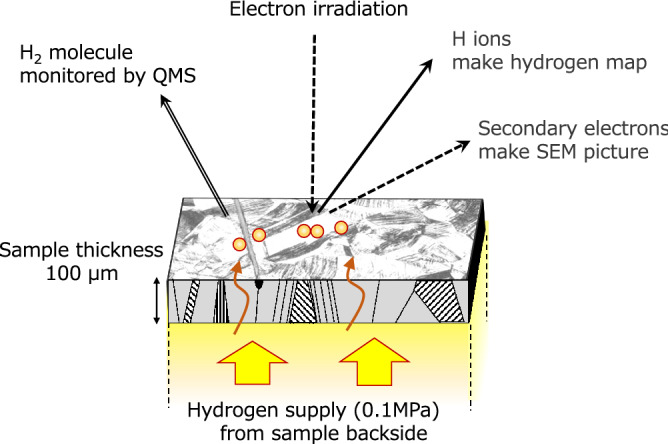
Schematic diagram of experiment set up of Operando Hydrogen Microscope. Hydrogen supplied from the one side of membrane sample, which is separating the experimental chamber into a hydrogen compartment and a ESD measurement compartment, permeates through the membrane to ESD measurement compartment. Permeated hydrogen is irradiated by electron beam, ionized and desorbed from the sample. The electron beam is also used to take a scanning electron microscope image (SEM image) with the emitted secondary electrons. The thermal desorbed hydrogen molecules can be monitored with a quadrupole mass spectrometer (QMS)^4^.

In our original “operando hydrogen microscope”^6,11^, the sample acts as a partition, separating the experimental chamber into two compartments: the bottom hydrogen compartment and the top ultrahigh vacuum (UHV) compartment. The sample set up is shown in Fig. 1, schematically. Hydrogen supplied at 0.1 MPa to the hydrogen compartment is adsorbed onto the bottom surface of the sample; it is then absorbed into the sample as an atom, diffuses within the sample, and exits into the UHV compartment. Hydrogen atoms permeating to the UHV surface are desorbed by electron irradiation from the surface as hydrogen ions or as hydrogen radicals. We use ESD to detect hydrogen ions, where the number of ions detected is proportional to the number of hydrogen atoms existing on the surface in the case that the surface is a metal with uniform elements. Electron energy of 1000 eV from the tungsten filament of a scanning electron microscope (JAMP10; JEOL Co., Tokyo, Japan) is used as the ESD electron source. Hydrogen maps are created continuously during the permeation experiment.

**Figure 2:**
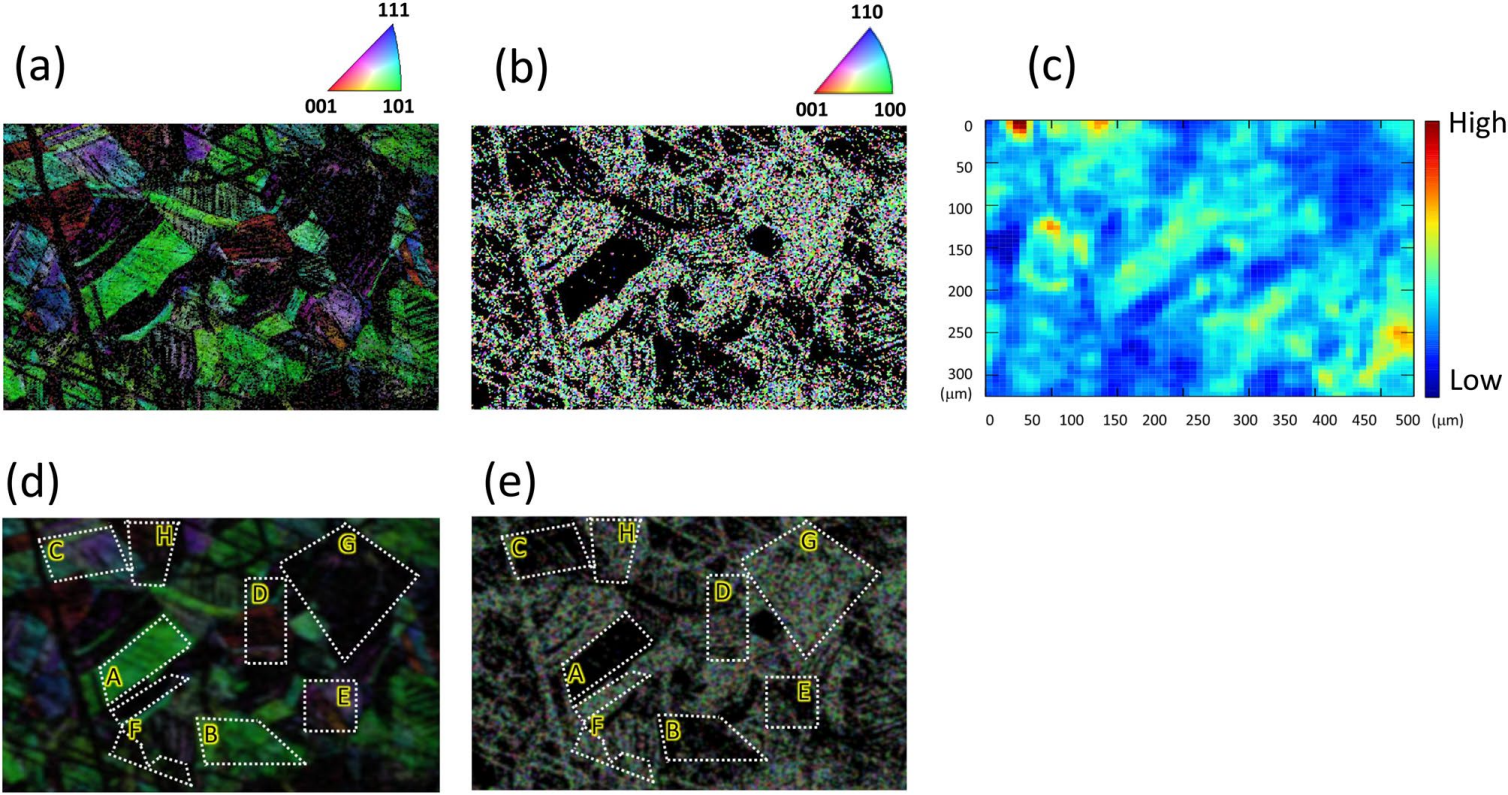
(**a**) Inverse pole figure (IPF) map for austenite in a sample of cold-worked SUS304 stainless steel. (**b**) Inverse pole figure map for martensite at the same position as (**a**). The colours in the IPF maps represent the crystal orientation when viewed from the normal direction (ND), shown in each upper right. (**c**) Distribution of hydrogen permeating through the sample. The colour bar indicates the hydrogen ion counts at each position. The ion counts were integrated over 65 h from the time that hydrogen was first introduced to the bottom of the sample. (**d**,**e**) Regions A to H (shown in dotted frames) from which characteristics were extracted. For clarity, they are overlaid on the EBSD images of (**b**) and (**c**), respectively. The sizes of each region A–H and the percentages of martensite in them are shown in Supplementary Table S2.

During the current experiment the temperature of the sample was kept at 473 K. The sample was SUS304 stainless steel composed of 18.18 wt% Cr; 8.41 wt% Ni; 1.23 wt% Mn; C, P, Si, and S below 1 wt% in sum total; and Fe for the balance. The steel was annealed for 1 h at 1100 °C to control the grain size to about 50–150 µm in diameter. At a sample thickness of 100 μm, hydrogen permeates the sample membrane from the bottom to the top surface through only one or a few grains. The annealed steel was rolled to 10% reduction in thickness at room temperature. When cold working meta-stable stainless steels such as SUS304, deformation-induced martensite phases with a large number of planar slip bands are formed^12^. For determination of microstructures in the sample, measurement by electron backscatter diffraction (EBSD) was performed using a field emission microscope (JSM7900F; JEOL Co.) equipped with a diffraction detector (DigiVew-V EBSD camera; AMETEK Co., Tokyo, Japan). In the EBSD measurement (Fig. 2), it was measured at a low magnification picture (× 150) in 2 μm steps. Since the acceleration voltage is 15 keV and the incident angle is 70°, the EBSD data reflect the information up to a depth of about 50 nm. The microstructure of the sample consisted of austenite and deformation-induced martensite phases. When estimating the martensite width, we used the EBSD data measured in 0.25 μm steps. In this dual-phase material, the chemical composition is the same as the base metal of SUS304, irrespective of the differences between the face-centred cubic structures (austenite) and body-centred cubic structures (martensite). A small amount of adsorbed oxygen was uniformly detected on the surface of the sample, but no localization in oxygen was observed on the sample. Therefore the hydrogen permeation behaviour for each phase reflects not the chemical composition but the crystal system.

Inverse pole figure (IPF) maps for austenite and martensite phases (structures) obtained by EBSD are shown in Fig. 2a,b, respectively. In the IPF maps, the normal direction (ND) was set normal to the sample surface. As shown in Fig. 2b, martensite phases appeared as lath-like structures that were too fine for their crystal orientations to be identified. No other structures, such as precipitates of solute elements, were detected at the surface in the area examined. Figure 2c shows hydrogen distribution measured by the operando hydrogen microscope in the area of Fig. 2a,b.

To explain the diffusion of hydrogen in mixed structures, we considered the behaviour of hydrogen in several small regions having different ratios of the two structures, taking the ratio as a variable. We selected eight regions, regions A–H shown on the IPF maps of austenite (Fig. 2d) and martensite (Fig. 2e). Region F is the sum of several small neighbouring regions.

## Diffusion coefficient and permeation flux of hydrogen in each region

In an experiment conducted over 65 h we obtained a set of 520 images of permeating hydrogen. The regions A–H in each of the 520 images were used to graph the time evolution of hydrogen ions present in each region (see Fig. 2 and Supplementary Fig. S1). The diffusion coefficient of each region was obtained by Fick’s law, from observing the time evolution of hydrogen ions desorbed by the ESD process. It was obtained by fitting curves with formula (1), (2) to the time evolution of ESD ion counts in the area of the regions^13^.

1 $$C(x,t) = C_{0} \left[ {1 - erf\left( {\frac{x}{{\sqrt {4Dt} }}} \right)} \right],$$

2 $$D = D_{0} \exp \left( { - \frac{{E_{d} }}{RT}} \right),$$

where *C* is concentration, *C*_0_ is a constant (concentration at steady state after equilibrium), *t* is time, *D* is diffusion coefficient, *D*_0_ is a constant (attempt frequency), *E*_*d*_ is activation energy, *x* is a distance from the bottom surface (maximum 100 µm), *R* is gas constant (8.31 J/K/mol), and *T* is temperature (473 K). The diffusion coefficients and other parameters (the values of *D*, *D*_*0*_, and *E*_*d*_) were obtained from the fitting results for each curve in each region.

**Figure 3:**
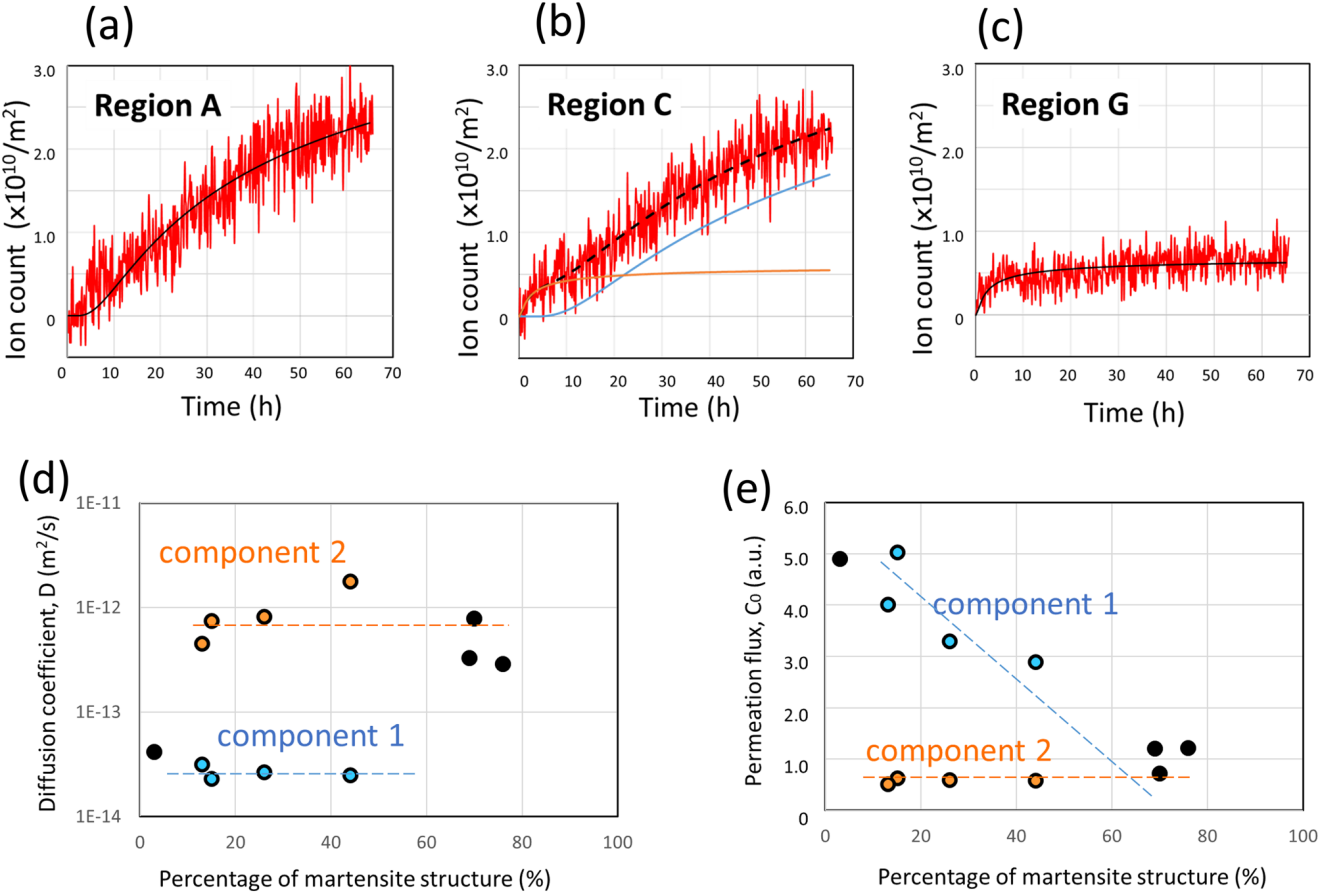
Ion counts plotted against time in each region. Counts are calibrated to the number per unit area. (**a**) Time evolution of ion counts desorbed in region A. The region size of ion counting was 5.3 × 10^−9^ m^2^, and 97% of the structure was austenite and 3% was martensite. (**b**) Time evolution of ion counts desorbed in region C. The region size was 6.4 × 10^−9^ m^2^, austenite and martensite structures accounted for 85% and 15%, respectively. (**c**) Time evolution of ion counts desorbed in region G. The region size was 1.6 × 10^−8^ m^2^, austenite and martensite structures were 30% and 70% respectively. Solid black lines in (**a**) and (**c**) are curves fitted using Fick’s law. Only the ion count line in (**b**) can be resolved into two fitting components. Component 1 (blue line) had a slow diffusion speed and component 2 (orange line) had faster one. (**d**) Diffusion coefficients of hydrogen in the eight local regions derived from the fitting results. They are plotted as a function of the ratio of martensite structure in each region. Solid black circles are single fitted results, such as shown in (**a**) and (**c**). Blue and orange circles are from resolved Component 1 and Component 2, such as in (**b**). (**e**) Permeation fluxes determined by fitting curves, which are the permeation fluxes after reaching steady-state hydrogen permeation, from the 0.1 MPa hydrogen compartment to the vacuum compartment through the 100 µm sample. For visual assistance, the features of Component 1 and Component 2 are marked with dashed lines, in (**d**) and (**e**).

Ion counts plotted against time in regions A–H revealed three different patterns, as exemplified by regions A, C, and G (Fig. 3). Time evolution of ion counts desorbed from region A, where 97% of the structure is austenite, could be fitted using Fick’s law with a single fitting curve (Fig. 3a). Time evolution of ion counts desorbed from region C, where 85% of the structure is austenite, could not be fitted by a single curve (Fig. 3b). It could be resolved into two fitting components: Component 1 (blue line) with a slow diffusion speed and Component 2 (orange line) with a faster diffusion speed. Ion counts in regions B, D, and E showed similar evolution to that in region C in that they each had two components (Supplementary Fig. S1). For region G, where only 30% is austenite, it could be fitted by a single fitting curve, which had fast diffusion speed but low ion counts (Fig. 3c). Regions F and H showed evolutions similar to that in region G (Supplementary Fig. S1). We suspected that the reason that only a single component appeared in regions with low austenite component (Fig. 3c) was because the Component 1 could not be seen owing to an insufficient number of hydrogen ions in the region. Therefore, we added together the hydrogen ions in the three regions of F, G, and H, and attempted a new fitting. Nevertheless, the Component 1 still could not be found.

Figure 3d shows the diffusion coefficients for regions A–H as a function of the percentage of martensite volume to total volume in the region. Region A, with a martensite ratio of 3% (97% austenite) shows one diffusion coefficient of Component 1 (solid black circle), corresponding to the single fitting curve in Fig. 3a. Regions B–E with a martensite fraction from 10 to 50% had two diffusion coefficients. These are due to two fitting curves, as shown in Fig. 3b. Diffusion coefficients from fitting Component 1 are shown as blue circles and those of Component 2 as orange circles. The three solid black circles around 70–80% are from regions F, G, and H, which each have a single diffusion coefficient (Component 2), as shown in Fig. 3c. Diffusion coefficients of Component 1 are close to that of the 97% austenite region, and the diffusion coefficients of Component 2 are close to the diffusion coefficients of martensite-dominant regions. What’s unusual here are the regions where the ratio of martensite volume to total volume is 70% or more. These regions still include austenite, but they did not show any properties of austenite diffusion. This is in contrast to austenite-rich regions, where even less than 15% of martensite maintained martensitic properties of diffusion.

As mentioned above, the number of ESD ions is proportional to the number of hydrogen atoms existing on a surface in the case that the surface is a metal with uniform elements. Comparing the number of hydrogen ions measured in each region is the same as comparing the flux of hydrogen that permeates each region. After sufficient time has passed from the start of hydrogen introduction, the flux of the region becomes constant as it reaches equilibrium, which is concentration *C*_0_. We compared the permeation flux of hydrogen in each region. Figure 3e plots the hydrogen permeation flux from the fitting results as a function of the percentage of martensite. The colours in the plots are the same as in Fig. 3d. The permeation flux of Component 2 looks to be independent from martensite percentage. On the other hand, that of Component 1 decreases as the martensite ratio increases. The three points around 70% are *C*_0_ from the single fitting curves of regions F–H. These three values are close to those of Component 2 but slightly larger.

From the hydrogen behaviour in Fig. 3d,e, we considered Component 1 to be austenite-derived hydrogen permeation and Component 2 to be martensite-derived hydrogen permeation. So, Fig. 3e can be read to mean that the permeation flux of the austenite component decreases as the martensite ratio increases while the permeation flux of martensite remains constant, independent of the proportion of martensite in the region.

Thus, hydrogen permeation cannot be explained simply in terms of a single microstructure. The diffusion path of hydrogen needs to be taken into account. The permeation flux of hydrogen in a region depends not only on the diffusion coefficient in the region through which hydrogen has permeated, but also on the solubility and concentration of hydrogen in the crystal structure. In the next section, we will consider the diffusion coefficient, solubility, and permeation flux together.

## Diffusion model and simulation

**Figure 4:**
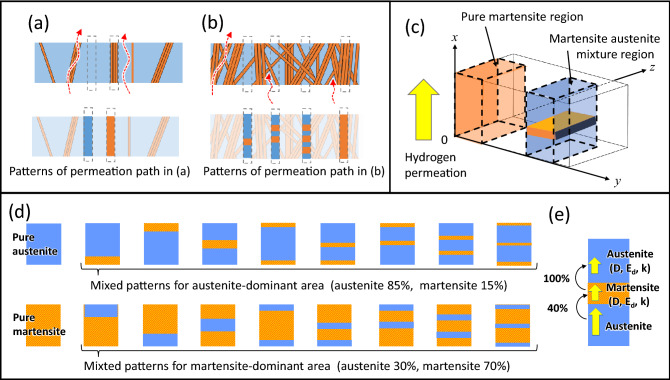
Schematic illustration of diffusion model of hydrogen permeation in a metal which has a dual structure. (**a**) Side view of an austenite-dominant region. There is a low percentage of martensite structures (orange) in the austenite structure (blue). Hydrogen permeates from bottom side to upper side (dashed red lines and arrows). (**b**) Side view of a martensite-dominant region. (**c**–**e**) Schematic images of mixed structures used for the simulation. (**d**) 18 block-patterns of austenite and martensite mixed structures are assumed for the simulation. Side views are shown as austenite (blue) and martensite (orange). For the austenite-dominant area, we chose values represented by region C (austenite 85% and martensite 15%). For the martensite-dominant area, we chose values represented by region G (austenite 30% and martensite 70%). Permeation path of hydrogen is from the bottom to top of each side view. (**e**) For the simulation we used diffusion coefficients (*D*), activation energies (*E*_*d*_), and solubilities (*k*) of austenite and martensite extracted from the literature, and used assumed values for the passage of hydrogen at the interface between phases.

We wrote that it is strange that hydrogen permeation through an austenite-dominant area was reflected by both austenitic and martensitic diffusion coefficients, while hydrogen permeating through a martensite-dominant area was reflected only by the martensitic diffusion coefficient. We explored the reason by considering the model shown in Fig. 4a,b. These figures schematically illustrate a cross-section through regions in which both austenite and martensite structures are mixed. It is assumed that hydrogen permeates upward from the bottom of the sample. We estimated by EBSD measurement that the average width of lath-like martensite phases in the sample was around 1.1 µm. When the martensite percentage is low, hydrogen supplied from the bottom surface will diffuse through only the austenitic phase or only the martensite phase as it penetrates to the upper surface (see the patterns of permeation paths in Fig. 4a). On the other hand, when the amount of sample volume related to the martensite phase, the austenite phase becomes fragmented by interspersed lath-like martensite phases (Fig. 4b), and hydrogen supplied from the bottom surface cannot diffuse only in the austenite phase to the upper surface. In this case, diffusion paths are either only through martensite or through an austenite–martensite mixture with several interfaces. This is why the diffusion coefficient of austenite did not appear as a component in regions where many martensite structures and few austenite structures co-exist. However, the sum of pure austenite and pure martensite could not quantitatively explain the permeation flux observed in regions with mixed structures. To explain this, it is necessary to also consider behaviour at the interfaces between martensite and austenite.

To quantitatively explain the observed permeation flux, we constructed a diffusion model having several types of martensite/austenite mixtures and simulated hydrogen permeation properties. The model assumes that hydrogen trapping processes can be ignored and that hydrogen diffusion through particular crystal structures such as austenite and martensite is based on Fick’s law. First, hydrogen diffusion through a single-crystal phase (pure austenite or pure martensite) was simulated to evaluate diffusion coefficients for hydrogen. The initial concentrations (*C*, at *x* = 0) for austenite and martensite were estimated by the solubilities of austenite and martensite^14,15^ with the hydrogen pressure in the hydrogen compartment, to which the bottom surface of the sample faced. We assume the units of each permeation pattern to be as shown in Fig. 4c,e. The hydrogen concentration at *x* = 0 and *t* = 0 is 0. Hydrogen diffusion coefficients *D* for austenite and martensite were obtained by Eq. (2), using *D*_0_ = 5.76 × 10^−7^ m^2^/s and *E*_*d*_ = 5.36 × 10^4^ J/mol for austenite^14^, and *D*_0_ = 2.82 × 10^−7^ m^2^/s and *E*_*d*_ = 3.44 × 10^4^ J/mol for martensite^15^. We considered martensite/austenite mixtures with one to four interfaces between phases (see Fig. 4d). We made eight patterns for a particular ratio of the austenite and martensite mixture. When the surface structure is austenite, the area could be pure austenite or it could be a mixed phase in which martensite is hidden beneath the surface austenite phase. Regarding the mixed conditions, an austenite-dominant model (austenite 85% and martensite 15%) and a martensite-dominant model (austenite 30% and martensite 70%) were considered. Assuming the structure of the inside regions to be either only austenite or a coexistence of austenite and martensite phases, we simulated the flux and velocity of hydrogen diffusion through the regions. The hydrogen concentration at the material surface facing the hydrogen gas (at a constant pressure) is proportional to the square root of the hydrogen pressure, *C* = *k*·*P*^1/2^. This is called Sievert’s law and is valid for solid solutions. Here, *k* is the solubility of hydrogen and *P* is the pressure on the exposed side (0.1 MPa). The solubility (*k*) is 54.1 mol/m^3^·MPa^1/2^ for the austenite phase and 21.7 mol/m^3^·MPa^1/2^ for the martensite. The solubility in martensite phase is about 40% of it in austenite phase. The hydrogen concentrations on the hydrogen supply side surface, *x* = 0, were calculated to be always 17.11 mol/m^3^ for austenite and 6.86 mol/m^3^ for martensite. Next, we made assumptions on the hydrogen transfer between phases. The transfer of hydrogen from the martensite phase, which has low hydrogen solubility, to the austenite phase, which has high hydrogen solubility, was assumed to be 100% of the hydrogen reaching the phase interface. On the other hand, the hydrogen transfer from the austenite phase to the martensite phase was considered to depend on the solubility ratio of each phase, and was assumed to be 40% of the hydrogen reaching the phase interface (see Fig. 4e).

**Figure 5:**
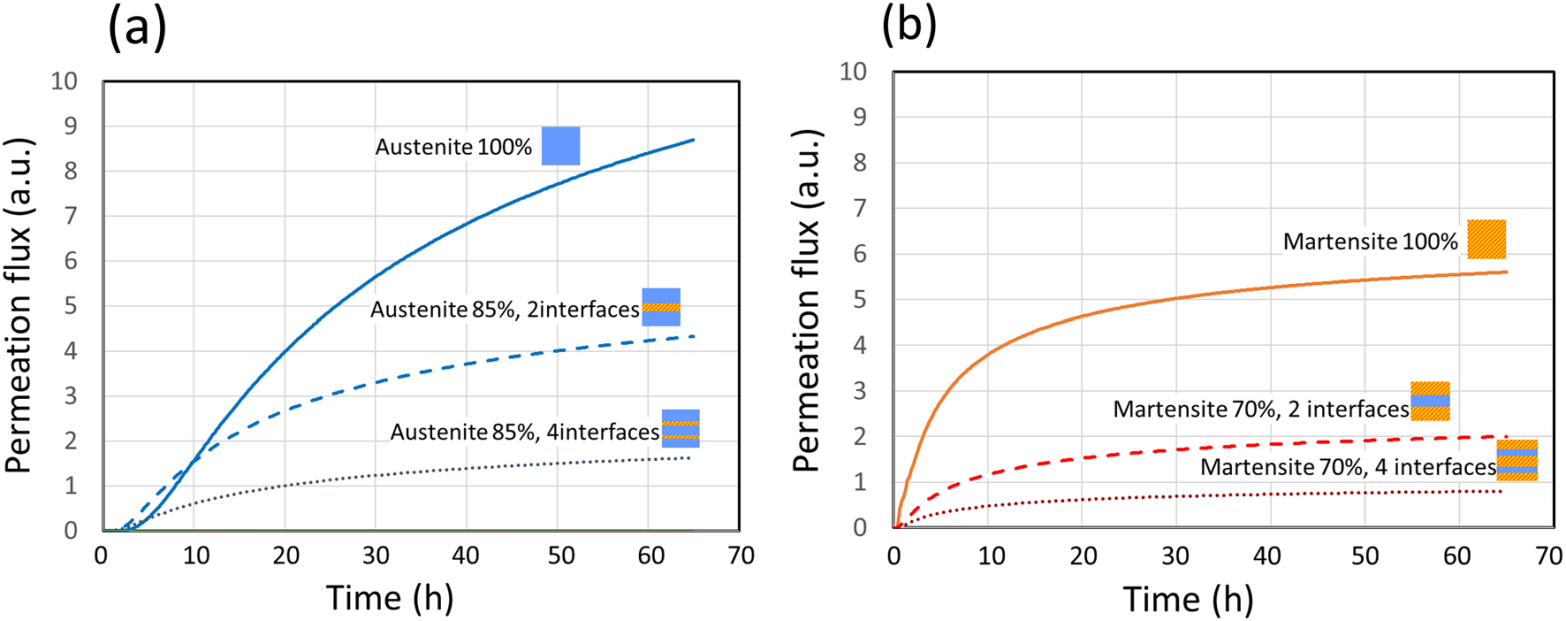
Simulated time evolution of the permeation flux of hydrogen through a 100-µm-thick sample with varied structure. (**a**) Hydrogen permeation through samples with austenite-dominant structure (85% austenite and 15% martensite) with two interfaces and four interfaces, and through pure austenite. (**b**) Hydrogen permeation through martensite-dominant structure (30% austenite and 70% martensite) with two interfaces and four interfaces, and through pure martensite.

The time evolution of hydrogen concentration at the surface was calculated for several model martensite/austenite structures. Figure 5a,b show the time evolutions of the permeation flux of hydrogen that has a diffusion path through austenite-dominant structures and through martensite-dominant structures, respectively. The diffusion flux of hydrogen is reduced by passing through a phase interface. Some of the lines calculated for mixtures with three interfaces and for four interfaces in Fig. 4d almost overlapped, and some for two interfaces and three interfaces overlapped, too (Supplementary Fig. S3). In such overlapped lines, the number of interfaces at which hydrogen was transported from austenite to martensite was the same. We have assumed the permeation at these interfaces to be 0.4, as mentioned above, corresponding to the difference in hydrogen solubility between austenite and martensite. This assumption has a big influence on the calculation result. Permeation was also found to depend on the structure facing the hydrogen supply; it was particularly small when the structure on the hydrogen supply side was martensite, even if the number of interfaces in the mixture was the same. By comparing the components derived from the results plotted in Fig. 3 and the calculated results in Fig. 5, Component 2 indicates a hydrogen diffusion path through 100% martensite. On the other hand, Component 1 indicates a hydrogen diffusion path through martensite, austenite, and the interfaces between the phases, which depends on the mixing ratio of the structures. This is a good explanation of the experimental result shown in Fig. 3e: Component 2 derived from martensite was almost constant, whereas Component 1 derived from austenite decreased with increasing martensite ratio.

The simulation in this paper considered only one-dimension vertical structure. In a three-dimension structure, there is the possibility that a martensite structure surrounding an austenite structure might create a hydrogen reservoir. More detailed hydrogen behaviour will be understood by creating a three-dimensional model in the future.

By measuring the time evolution of hydrogen visualized at the sample surface, it has become possible to infer the hydrogen diffusion path inside of the sample. Despite the experimental method not directly measuring the structure within the sample, this information can be inferred from the diffusion behaviour of hydrogen. Hydrogen permeation can be utilized for a structural analysis of the sample.

## Summary

We used an operando hydrogen microscope, which combines electron-stimulated desorption (ESD) and a hydrogen supply system, for hydrogen visualization, and interpreted the local diffusion behaviours of hydrogen from the time evolution of hydrogen visualized at the surface. The sample we used was cold-worked stainless steel having both austenite and martensite structures; the martensite/austenite ratio was different in each region of the sample, whereas the chemical composition was uniform over the sample. The hydrogen diffusion path, through both martensite structures and austenite structures and across the interfaces between them, could be inferred from the time evolution of hydrogen permeation in multiple regions. We proposed a model of hydrogen diffusion in a dual-structure material and examined the validity of the model by a simulation that took into account the variations in the numbers of interfaces between martensite and austenite.

This is the first study to directly observe the dynamic behaviour of local hydrogen in multiple regions with different structures. Not only will it be useful for researching materials in the hydrogen economy but it might also lead to a new research method utilizing hydrogen permeation for structural analysis of materials.

## Supplementary Information

Supplementary Information 1.

Supplementary Information 2.

Supplementary Information 3.
