# Supplementary material for: Model of local hydrogen permeability in stainless steel with two coexisting structures
Source: Sci Rep. 2021 Apr 20;11:8553. doi: 10.1038/s41598-021-87727-5 (PMC8058332; doi:10.1038/s41598-021-87727-5)

Model of local hydrogen permeability in stainless steel with two coexisting structures

Akiko N. Itakura

Supplementary information S1

Time evolution of ion counts desorbed from regions A to H. The diffusion coefficients and permeation fluxes derived from the fitting results for each region are summarized in Figures 2d and 2e.

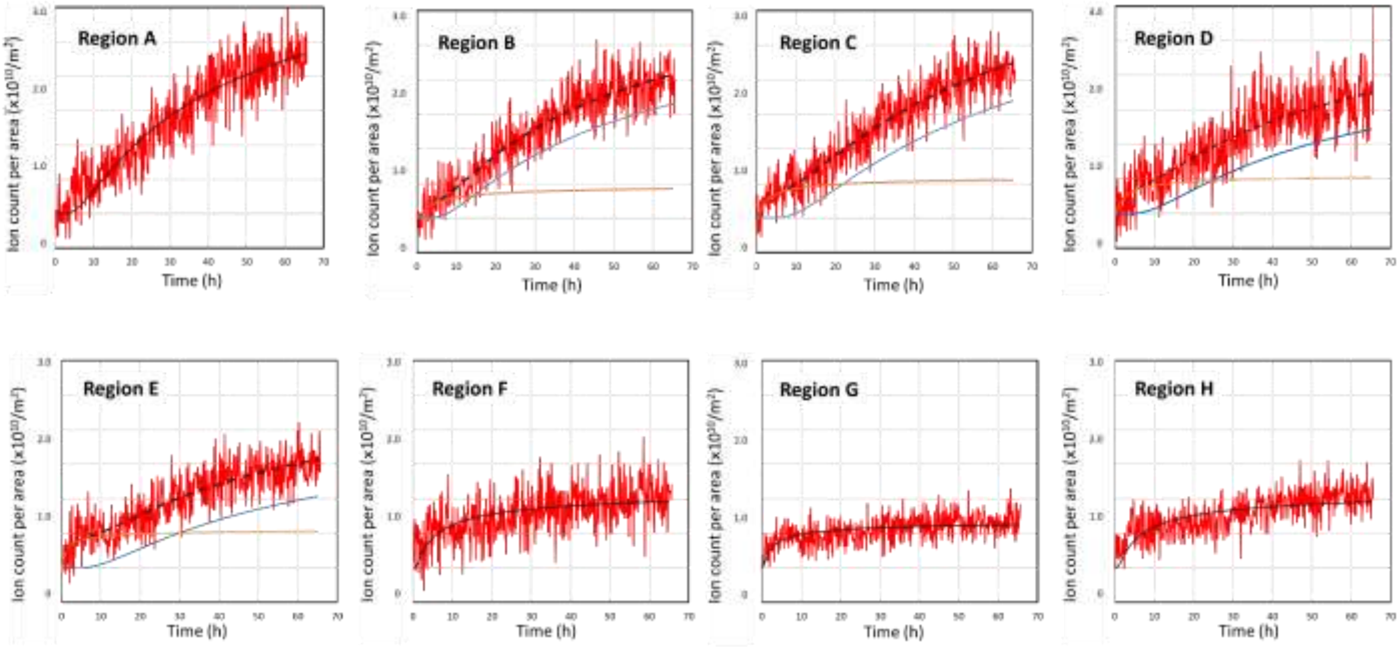

Supplement: Supplementary file 1 — Supplementary Information 1. [file 41598_2021_87727_MOESM1_ESM.pdf]
