# Supplementary material for: Model of local hydrogen permeability in stainless steel with two coexisting structures
Source: Sci Rep. 2021 Apr 20;11:8553. doi: 10.1038/s41598-021-87727-5 (PMC8058332; doi:10.1038/s41598-021-87727-5)
Supplement: Supplementary file 2 — Supplementary Information 2. [file 41598_2021_87727_MOESM2_ESM.pdf]

Model of local hydrogen permeability in stainless steel with two coexisting structures

Akiko N. Itakura

Supplementary information S2

The sizes of regions A to H and the percentage of martensite in the regions are shown in Table S2.

Region	Size ($\times 10^{-9}$ m^2)	Percentage of martensite (%)
A	5.3	3
B	7.2	13
C	6.4	15
D	4.0	26
E	7.4	44
F	4.2	69
G	15.6	70
H	7.8	76
