# Supplementary material for: Model of local hydrogen permeability in stainless steel with two coexisting structures
Source: Sci Rep. 2021 Apr 20;11:8553. doi: 10.1038/s41598-021-87727-5 (PMC8058332; doi:10.1038/s41598-021-87727-5)

Model of local hydrogen permeability in stainless steel with two coexisting structures

Akiko N. Itakura

Supplementary information S3.

Some of simulated lines of time evolution of the permeation flux overlapped. Two lines are added to Figure 4a. In such overlapped lines, the number of interfaces at which hydrogen was transported from austenite to martensite was the same.

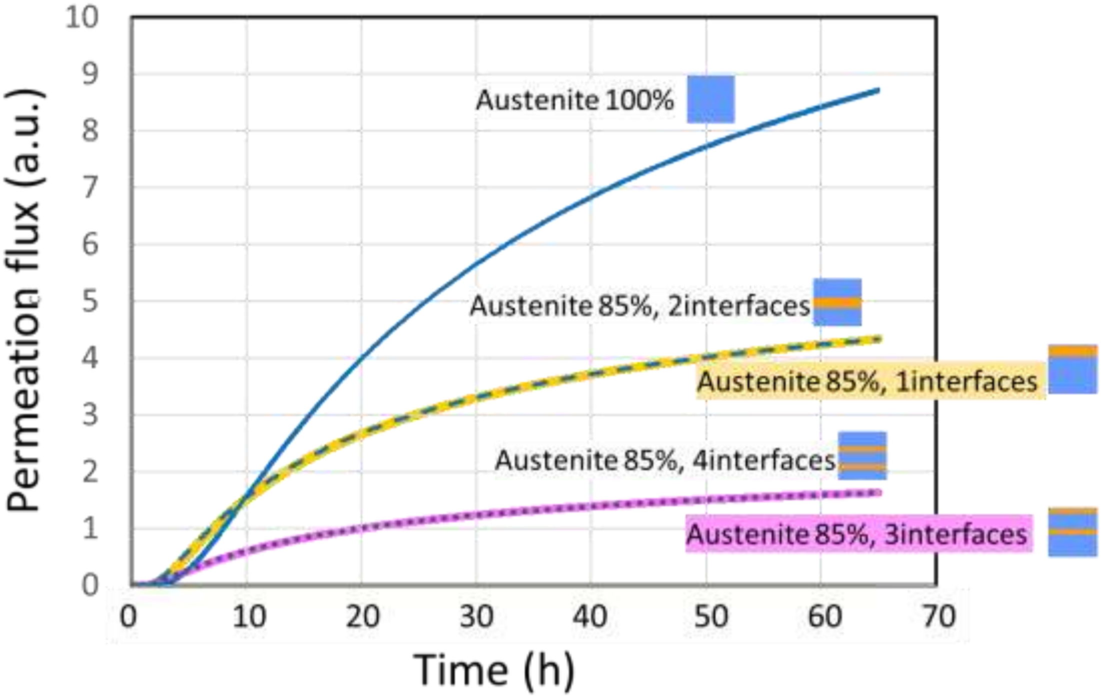

Supplement: Supplementary file 3 — Supplementary Information 3. [file 41598_2021_87727_MOESM3_ESM.pdf]
